# CD19‐targeting chimeric antigen receptor T‐cell therapy is safe and effective for intra‐cardiac B cell non‐Hodgkin lymphoma

**DOI:** 10.1002/jha2.1020

**Published:** 2024-10-17

**Authors:** Daniel H. Chen, Maeve O'Reilly, Kathleen P. Cheok, Ryan Low, Rajesh Puranik, Samuel Clark, John Malcolm Walker, Charlotte Manisty, Arjun K. Ghosh, Claire Roddie

**Affiliations:** ^1^ University College Hospital University College London Hospitals NHS Foundation Trust London UK; ^2^ Hatter Cardiovascular Institute University College of London London UK; ^3^ Prince of Wales & St George Hospitals South East Sydney Local Health District Sydney Australia; ^4^ University College London Cancer Institute University College London London UK; ^5^ Royal Prince Alfred Hospital Sydney Australia; ^6^ Charles Perkins Centre University of Sydney Sydney Australia; ^7^ Barts Heart Centre St Bartholomew's Hospital Barts Health NHS Trust London UK

**Keywords:** B cell non‐Hodgkin lymphoma, cardio‐oncology, cardiovascular adverse events, cardiovascular diseases, chimeric antigen receptor T‐cell

## Abstract

**Introduction:**

Chimeric antigen receptor T‐cell (CAR‐T) therapy is highly effective in B‐cell blood cancers, but there is limited data on its safety and efficacy in intra‐cardiac lymphoma, due to the potential risks of cardiotoxicity and pseudo‐progression.

**Discussion:**

We discuss four high‐risk cases that were managed with a multi‐disciplinary approach, including baseline cardiac risk assessment and surveillance with multimodal cardiac imaging and serum cardiac biomarkers, elective supportive care in the intensive care unit, and early treatment of cytokine release syndrome.

**Conclusion:**

CAR‐T therapy can be effective and safe in the treatment of B‐cell blood cancers with intra‐cardiac disease.

While CD19‐targeting chimeric antigen receptor T‐cell (CD19CAR‐T) therapy with axicabtagene ciloleucel (Axi‐cel) [1] and brexucabtagene autoleucel (Brexu‐cel) for relapsed/refractory (r/r) large‐B‐cell‐lymphoma (LBCL), and mantle cell lymphoma (MCL), respectively [2] is highly effective, cardiac decompensation during cytokine release syndrome (CRS) is a life‐threatening complication [3, 4]. For patients with intra‐cardiac lymphoma, potentially heightened risks of CRS‐associated cardiotoxicity and intra‐cardiac ‘pseudo‐progression [5] leading to gross cardiac dysfunction/rupture [6, 7] are a serious concern. Currently, there is limited data on the safety and efficacy of CAR‐T therapy in intra‐cardiac lymphoma with the exception of a single case report of CAR‐T leading to cardiogenic shock [8].

Here we describe four cases of CAR‐T therapy for intra‐cardiac B‐NHL, highlighting efficacy and toxicity (including ‘pseudo‐progression’) in this high‐risk group.

Patients with r/r intra‐cardiac lymphoma following failure ≥2 therapy lines referred for licensed CD19CAR‐T therapy were recruited into the CARTCO (NCT05130489) study at the University College London Hospital. CARTCO incorporates baseline electrocardiography (ECG), transthoracic echocardiography (TTE), cardiac magnetic resonance imaging (CMR), and cardiac biomarkers (Troponin‐T [Tn]; *N*‐terminal‐pro‐B‐type‐natriuretic peptide [NT‐proBNP]), repeated at Day 7, Day 28, and Month 3 [3]. The ASTCT CRS and ICANS grading system was used [9]. Multi‐disciplinary management (haematologists/intensivists/cardio‐oncologists), and pre‐planned intensive peri‐CAR‐T inpatient surveillance were employed.

## 1 CASE 1

**FIGURE 1:**
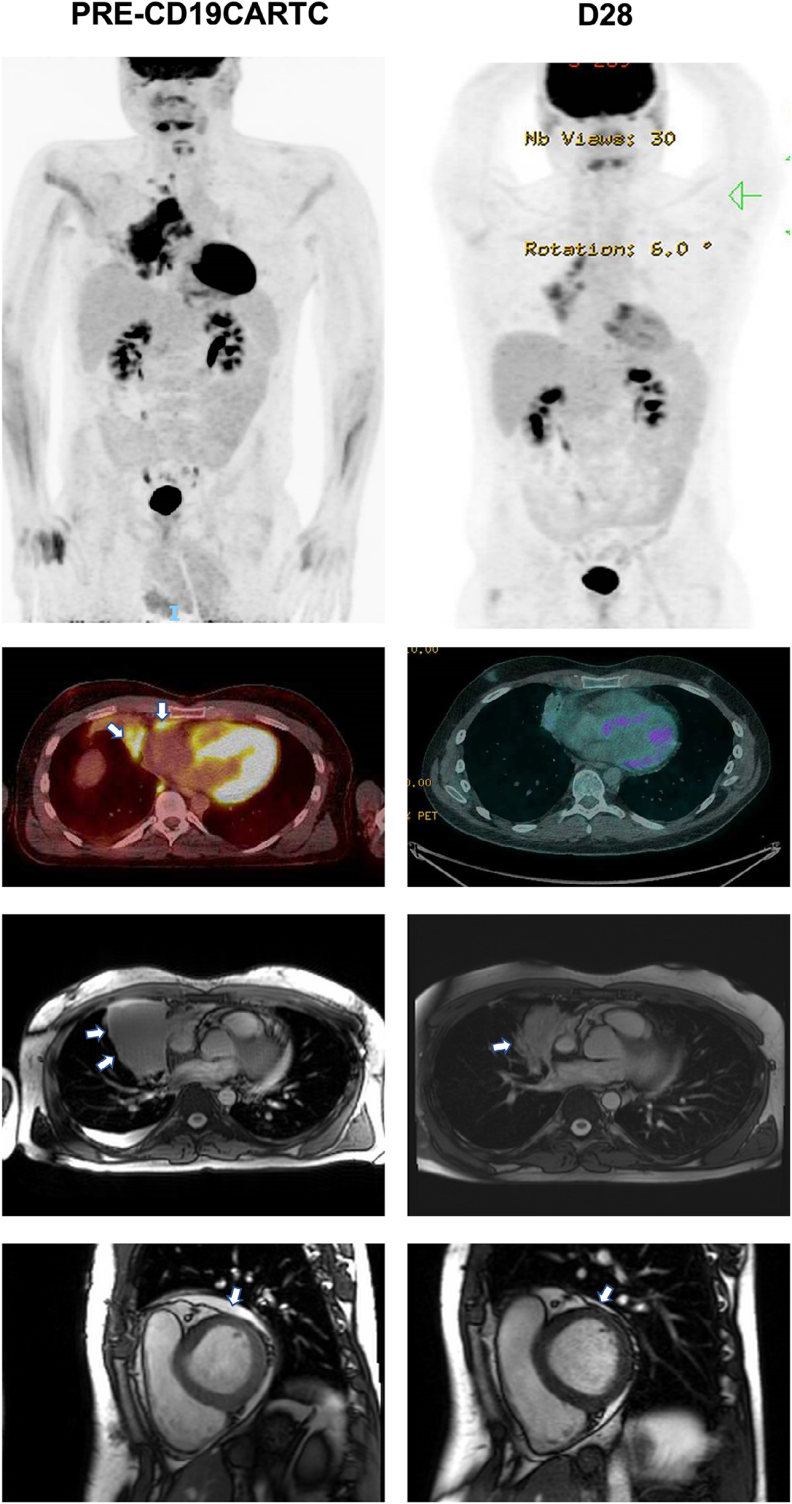
A 24‐year‐old male with relapsed/refractory (r/r) large‐B‐cell‐lymphoma (LBCL) was treated with CD19‐targeting chimeric antigen receptor T‐cell (CD19CAR‐T). Computed tomography‐positron emission tomography (CT‐PET) demonstrated a 77 × 58 mm mass at the right hilum adjacent to the right atrium, with disease extension into the pericardium which was FDG‐avid. Small pericardial effusion (8 mm) on cardiac magnetic resonance imaging (CMR); no endomyocardial tumor invasion. CMR at day 28 post‐CD19CAR‐T Reduction in the right hilar mass on CMR at day 28 post‐CD19CAR‐T and Deauville 4 response on PET‐CT.

A 25‐year‐old male received axi‐cel for a 77 × 58 mm right hilar LBCL mass invading the right atrium, with an 8 mm pericardial effusion on CMR. TTE demonstrated normal systolic function (ejection fraction [EF] = 60%; normal > 55%) without constriction. ECG and cardiac biomarkers were normal. Grade 1 (G1) CRS developed on Day 2. On Day 5, atrial fibrillation (AF) (140–150 bpm) and hypotension (80/50 mmHg) developed, consistent with G2 CRS, resolving with tocilizumab, corticosteroids, and bisoprolol. Day 28 CMR showed a smaller right hilar mass, no myocardial edema, and preserved left ventricle (LV) function. Positron emission tomography‐computed tomography (PET‐CT) showed a Deauville 4 response, ongoing at month 3 (Figure 1).

## 2 CASE 2

**FIGURE 2:**
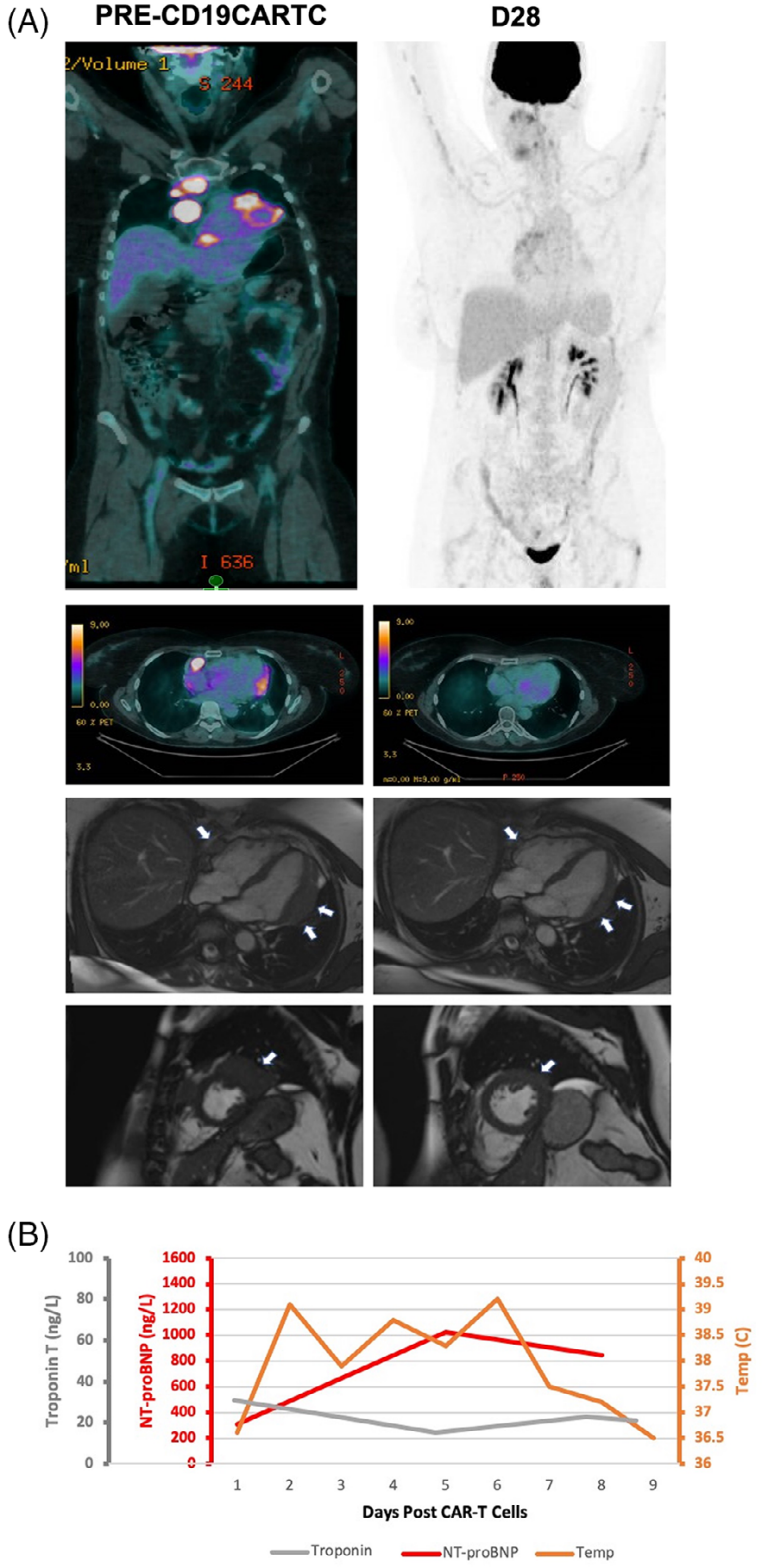
(A) A 46‐year‐old female with relapsed/refractory (r/r) large‐B‐cell‐lymphoma (LBCL) underwent treatment with CD19‐targeting chimeric antigen receptor T‐cell (CD19CAR‐T). FDG‐avid disease within the heart on computed tomography‐positron emission tomography (CT‐PET) at baseline, corresponding to multifocal myocardial and pericardial lymphomatous infiltration on cardiac magnetic resonance imaging (CMR) including an intramyocardial lateral left ventricle (LV) mass measuring 24 × 31 mm. CT‐PET at day 28 post‐CD19CAR‐T demonstrated a Deauville 4 response and regression of the intramyocardial LV wall mass without evidence of myocardial edema on CMR. (B) Fevers and grade 1 CRS were associated with an elevated NT‐proBNP; TnT levels did not change.

A 46‐year‐old female received axi‐cel for multifocal myocardial/pericardial LBCL with an intramyocardial mass (24 × 31 mm) and invasion/compression of the superior vena cava (Figure 2A). TTE showed normal LV function (EF = 55%) without constriction. ECG was normal. Tn‐T (36 ng/L; normal < 14 ng/L) and NT‐proBNP (307 ng/L; normal < 125 ng/L) were mildly elevated. The patient was electively admitted to the ICU on Day 0 for cardiac monitoring. On Day 1, G1 CRS was developed, and tocilizumab was infused. TTE showed reduced LV function (EF = 47%) which was normalized by Day 3 (EF = 57%). NT‐proBNP peaked on Day 4 but declined to 845 ng/L on Day 7 (Figure 2B). Further arrhythmias and cardiovascular events were not observed. Day 28 PET‐CT and CMR showed a Deauville 4 response (ongoing, month 6) and regression of the intramyocardial mass (Figure 2A).

## 3 CASE 3

**FIGURE 3:**
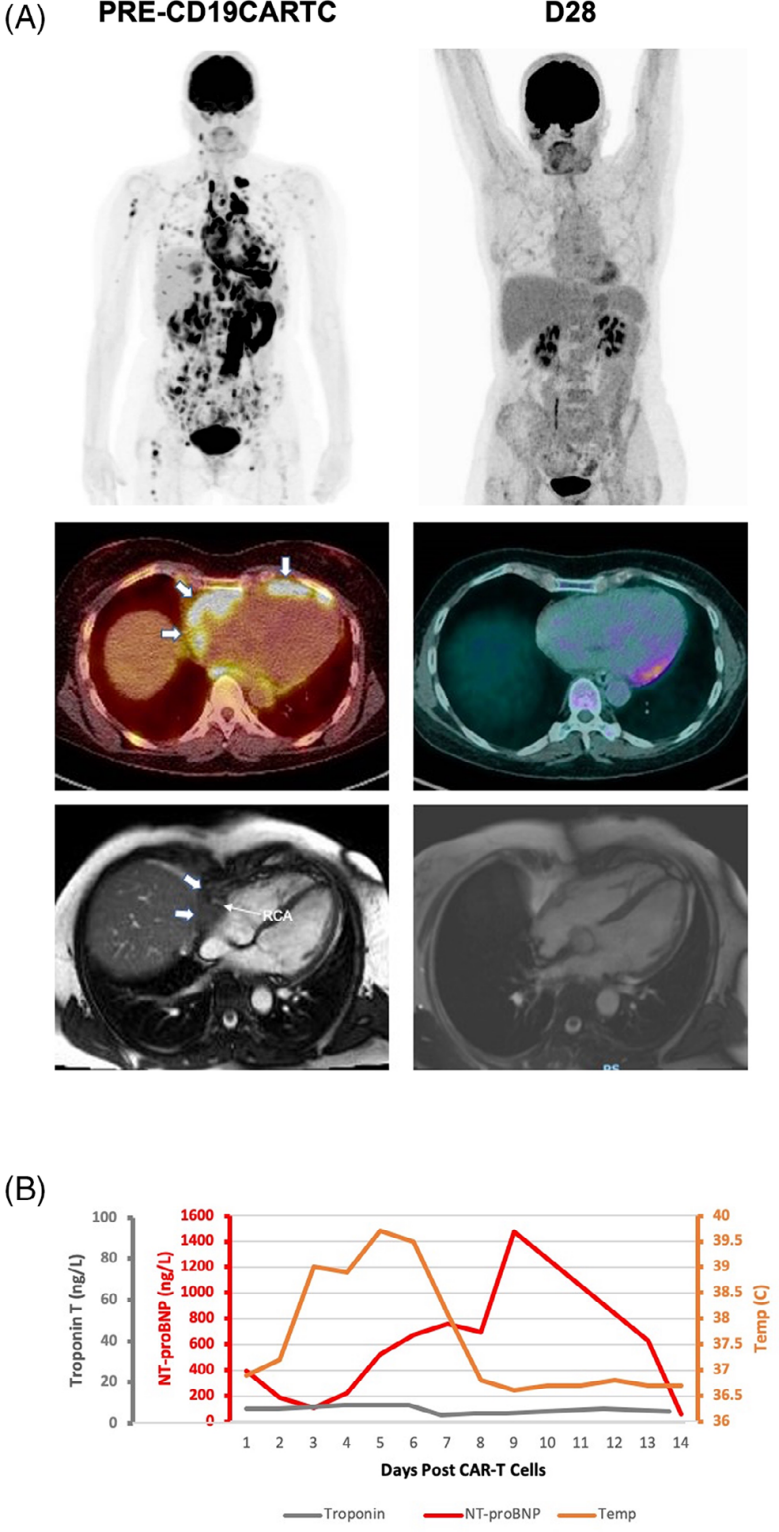
(A) A 50‐year‐old female with pericardial large‐B‐cell‐lymphoma (LBCL) was treated with CD19‐targeting chimeric antigen receptor T‐cell (CD19CAR‐T). Pericardial effusion on pre‐treatment cardiac magnetic resonance imaging (CMR) and a pericardial mass infiltrating the RV and RA with encasement of the right coronary artery, corresponding to FDG‐avid disease on computed tomography‐positron emission tomography (CT‐PET). CT‐PET assessment at day 28 demonstrated a Deauville 3 complete response and a significant reduction in the size of the pericardial mass on CMR. (B) Post CD19CAR‐T, she developed grade 2 CRS and LVSD associated with a rise in NT‐proBNP; TnT levels did not change.

A 50‐year‐old female received an axi‐cel for LBCL invading the right atrium/ventricle and a pericardial effusion (Figure 3A). TTE showed systolic dysfunction (EF = 54%) without constriction. ECG and cardiac biomarkers were normal (Figure 3B). The patient was electively admitted to the ICU on Day 0 for cardiac monitoring. G2 CRS emerged on Day 2 with fever, hypotension (80/40 mmHg), anterolateral T‐wave inversion on ECG, global, moderate systolic dysfunction (EF = 44%) on TTE without pericardial effusion/tamponade (Figure 3A), and was refractory to tocilizumab but responded to corticosteroids. The patient had no further cardiac complications. ECG changes normalized by Day 7 with partial recovery of systolic function (EF = 50%). NT‐proBNP peaked on Day 8 (Figure 3B). Day 28 PET‐CT and CMR showed a Deauville 3 response, recovery of systolic function (EF = 58%), and a reduction in the pericardial mass, ongoing at month 3 (Figure 3A), with normalized NT‐proBNP (Figure 3B).

## 4 CASE 4

**FIGURE 4:**
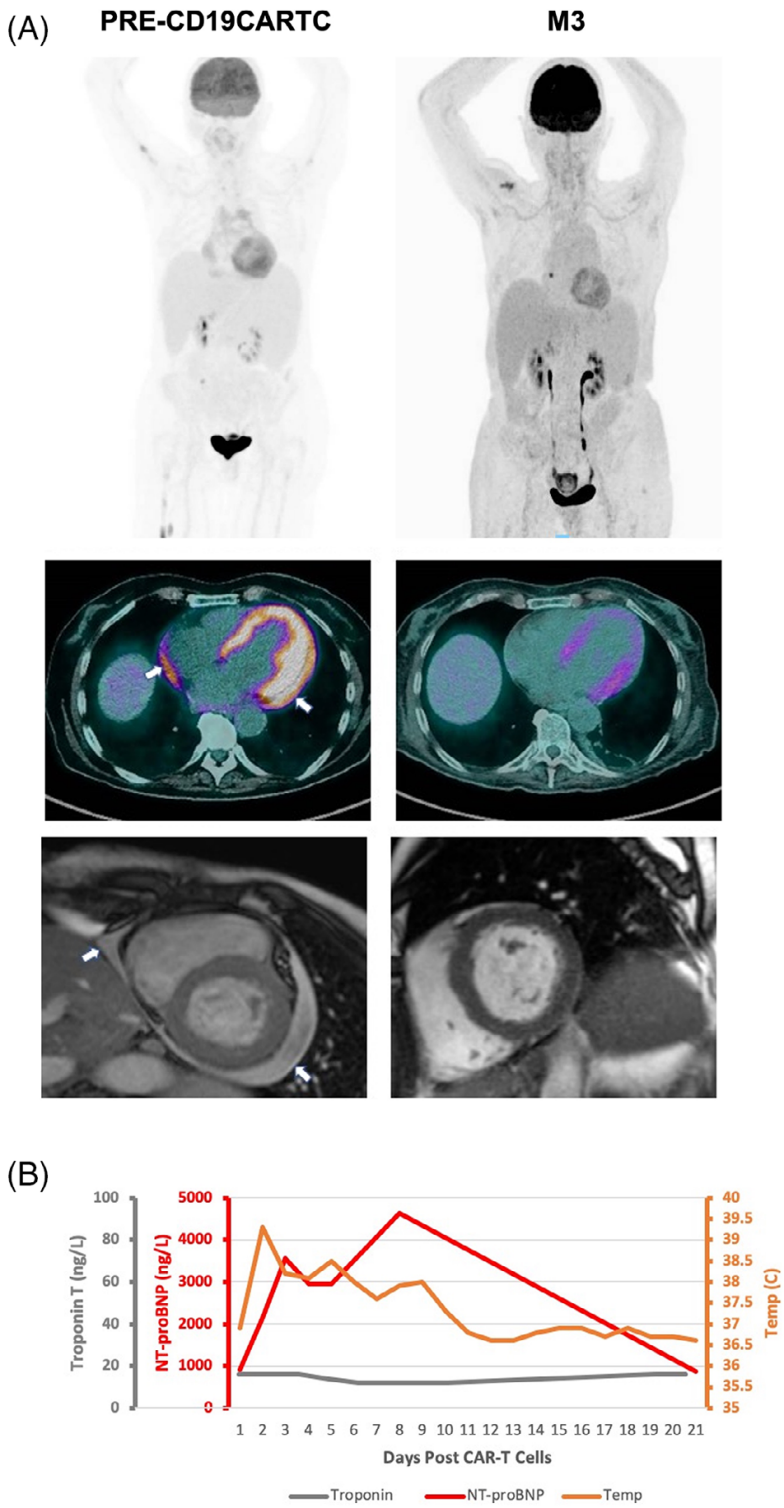
(A) A 69‐year‐old female with relapsed/refractory (r/r) mantle cell lymphoma with pericardial involvement received CD19‐targeting chimeric antigen receptor T‐cell (CD19CAR‐T). Moderate, loculated pericardial effusion on pre‐treatment cardiac magnetic resonance imaging (CMR) consistent with metabolically active disease on computed tomography‐positron emission tomography (CT‐PET). Complete resolution of the pericardial effusion was seen on CT‐PET and CMR at month 3. (B) A paroxysm of AF during LD but prior to CD19CAR‐T resulted in pulmonary edema and NT‐proBNP elevation. Following CD19CAR‐T, she developed fevers and grade 1 CRS along with LVSD resulting in further NT‐proBNP elevation. TnT remained unchanged.

A 69‐year‐old female received brexu‐cel for MCL involving the pericardium without tamponade/constriction. CMR and ECG showed anthracycline‐induced cardiomyopathy with borderline systolic dysfunction (EF = 55%), diffuse fibrosis, and inferolateral T wave inversion (Figure 4A). Baseline cardiac biomarkers were elevated (Tn‐T 22 ng/L; normal <14 ng/L. NT‐proBNP 919 ng/L; normal <125 ng/L) (Figure 4B). Following lymphodepletion (LD) and prior to Brexu‐cel, she developed AF (rate,115–120 bpm) with raised NT‐proBNP (2000 ng/L), which resolved with bisoprolol. On Day 2 she developed fever, paroxysms of AF (120–130 bpm), and hypoxia (FiO2,1L/min), with pulmonary edema. This was resolved with diuretics, bisoprolol, and tocilizumab. Day 3 TTE showed reduced systolic function (EF = 40%–50%). NT‐proBNP peaked at 4,642 ng/L (normal <125 ng/L) on Day 7 (Figure 4B), but no further cardiovascular events were observed thereafter. Day 28 and month 3 PET‐CT and CMR showed resolution of pericardial disease, recovering myocardial edema, and normalized LV function (EF = 60%). NT‐proBNP returned to baseline (876 ng/L; normal <125 ng/L).

CAR‐T‐associated cardiotoxicity affects 12–21% of LBCL patients [3], but risks are poorly characterized in intra‐cardiac lymphoma beyond a single case report describing cardiogenic shock and multiorgan failure [8]. Here we review four patients with intra‐cardiac lymphoma treated with CAR‐T and show that it is safe and effective when delivered by an expert multidisciplinary team, incorporating early elective ICU admission for monitoring and comprehensive baseline assessment of cardiac anatomy/function/biomarkers, tumor bulk, arrhythmias, and fluid status.

CMR is the gold standard for myocardial characterization [10] and was used here to assess intra‐cardiac disease sites [11]. Elevated Tn, indicative of myocardial injury, and NT‐proBNP, a sensitive marker for congestive heart failure [12] may be used to predict cardiac risk and detect subclinical disease [13]. In systemic lymphoma, high Tn post‐CAR‐T correlates with higher rates of > G2 CRS and new systolic dysfunction [14], and high baseline NT‐proBNP correlates with higher rates of > G2 CRS [15]. Prognostic implications for these biomarkers in intra‐cardiac lymphoma will require further validation.

**FIGURE 5:**
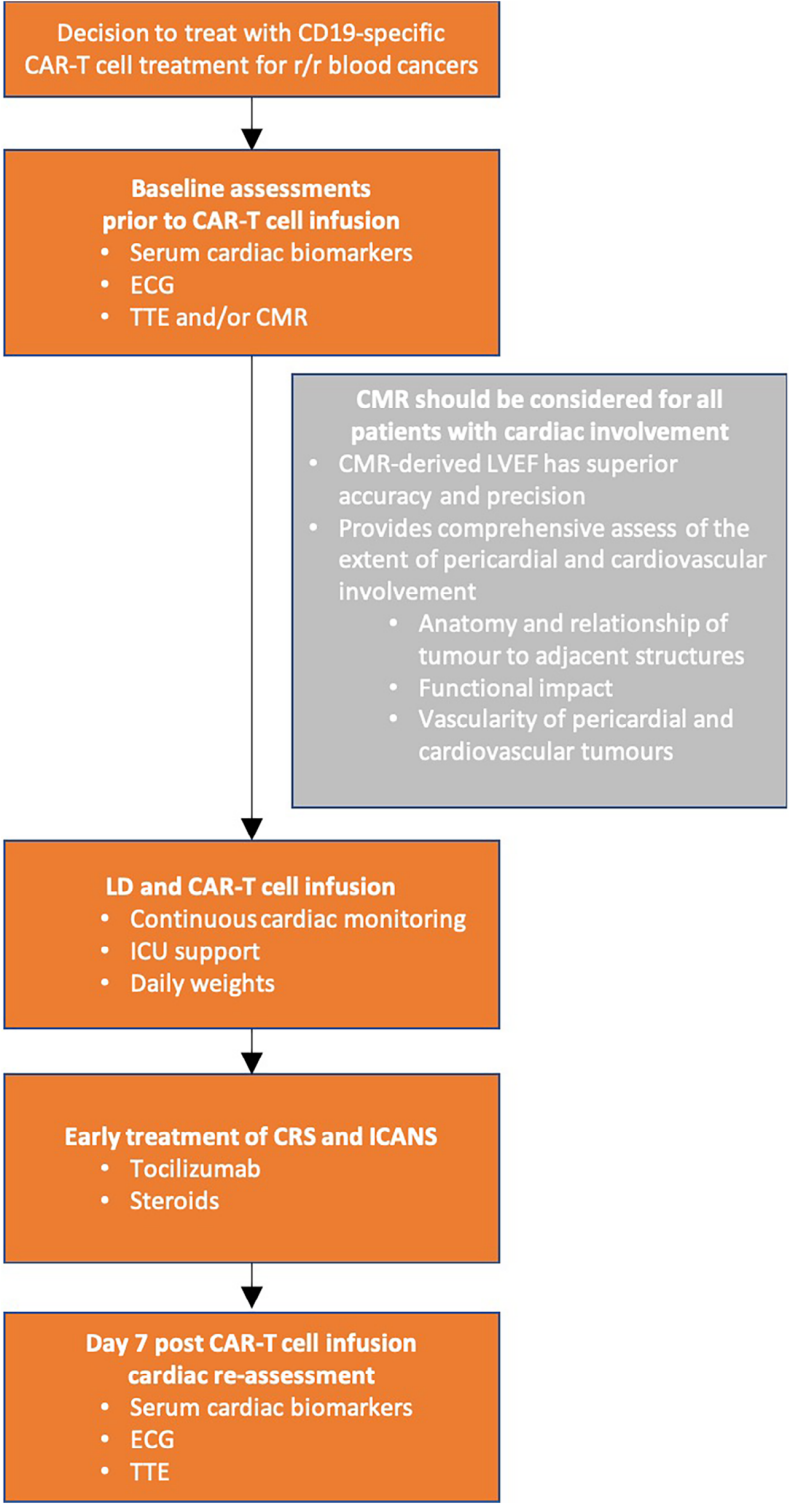
Management algorithm in patients receiving CD19‐targeting chimeric antigen receptor T‐cell (CD19CAR‐T) for B‐NHL with cardiac involvement—this includes careful pre‐treatment cardiac assessment with serum biomarkers, cardiac imaging, and electrocardiography (ECG), close surveillance during treatment and continuous monitoring within ICU, and early treatment of low‐grade CRS with tocilizumab and corticosteroids where appropriate.

Here, all four patients developed cardiotoxicity. Atrial arrhythmia in patient 1 prompted a shift in practice towards ICU‐based cardiac surveillance for 7–10 days post‐CAR‐T and earlier treatment of CRS with tocilizumab/corticosteroids. In contrast to Koch et al [8], none of these patients experienced life‐threatening cardiotoxicity which may be due to lower disease burden following bridging, elective pre‐CAR‐T management of tamponade/constriction, and pre‐emptive ICU‐based cardiac monitoring. A proposed management algorithm is outlined in Figure 5.

In summary, despite the potential for heightened cardiotoxicity, CAR‐T therapy can be delivered safely and effectively for intra‐cardiac lymphoma. We suggest that these high‐risk patients are managed by an expert multidisciplinary team, with a careful baseline assessment of cardiac risk using imaging/biomarkers, intensive peri‐CAR‐T inpatient surveillance, and early intervention for CRS. As CAR‐T indications expand into solid tumors and autoimmunity, robust, proactive cardiotoxicity management guidelines [13] will be needed to refine risk stratification toward optimal resource utilization and patient outcomes.

## AUTHOR CONTRIBUTIONS

Claire Roddie and Daniel H Chen conceived the project, devised the algorithm, analyzed the data, and wrote the manuscript. Maeve O'Reilly, Kathleen P. Cheok, Ryan Low, Rajesh Puranik, and Samuel Clark collected data and edited the manuscript; J. Malcolm Walker, Charlotte Manisty, and Arjun K. Ghosh devised the algorithm, analyzed data, and edited the manuscript.

## CONFLICT OF INTEREST STATEMENT

Dr. Claire Roddie received honoraria from Kite Gilead, Novartis, Autolus, and Bristol Myers Squibb. Dr. Maeve O'Reilly has served on advisory boards and received honoraria from Kite/Gilead, Novartis, and Janssen. Dr. Daniel Chen received honoraria from Novartis. The remaining authors have nothing to disclose.

## FUNDING INFORMATION

The authors received no specific funding for this work.

## ETHICS STATEMENT

All patients described in this case series provided consent for publication.

## PATIENT CONSENT STATEMENT

The authors have confirmed patient consent statement is not needed for this submission.

## CLINICAL TRIAL REGISTRATION

The authors have confirmed clinical trial registration is not needed for this submission.

## Data Availability

All data are available from the corresponding author upon reasonable request.
